# Thermosensitive PLGA–PEG–PLGA Hydrogel as Depot Matrix for Allergen-Specific Immunotherapy

**DOI:** 10.3390/pharmaceutics14081527

**Published:** 2022-07-22

**Authors:** Sonja Heine, Antonio Aguilar-Pimentel, Dennis Russkamp, Francesca Alessandrini, Valerie Gailus-Durner, Helmut Fuchs, Markus Ollert, Reinhard Bredehorst, Caspar Ohnmacht, Ulrich M. Zissler, Martin Hrabě de Angelis, Carsten B. Schmidt-Weber, Simon Blank

**Affiliations:** 1 Center of Allergy and Environment (ZAUM), Technical University of Munich, School of Medicine and Helmholtz Center Munich, German Research Center for Environmental Health, 85764 Munich, Germany; sonja.heine@helmholtz-muenchen.de (S.H.); dennis.russkamp@web.de (D.R.); franci@helmholtz-muenchen.de (F.A.); caspar.ohnmacht@helmholtz-muenchen.de (C.O.); ulrich.zissler@tum.de (U.M.Z.); csweber@tum.de (C.B.S.-W.); 2 Institute of Experimental Genetics, German Mouse Clinic, Helmholtz Center Munich, German Research Center for Environmental Health, 85764 Neuherberg, Germany; aguilar@helmholtz-muenchen.de (A.A.-P.); gailus@helmholtz-muenchen.de (V.G.-D.); hfuchs@helmholtz-muenchen.de (H.F.); hrabe@helmholtz-muenchen.de (M.H.d.A.); 3 Department of Infection and Immunity, Luxembourg Institute of Health (LIH), 4354 Esch-Sur-Alzette, Luxembourg; markus.ollert@lih.lu; 4 Department of Dermatology and Allergy Center, Odense Research Center for Anaphylaxis, University of Southern Denmark, 5000 Odense, Denmark; 5 Institute of Biochemistry and Molecular Biology, University of Hamburg, 20146 Hamburg, Germany; reinhard.bredehorst@chemie.uni-hamburg.de; 6 Chair of Experimental Genetics, School of Life Science Weihenstephan, Technical University of Munich, 85354 Freising, Germany; 7 German Center for Diabetes Research (DZD), 85764 Neuherberg, Germany

**Keywords:** allergen-specific immunotherapy, allergic asthma, antigen release, hydrogel, delivery system, depot matrix

## Abstract

Allergen-specific immunotherapy (AIT) is the only currently available curative treatment option for allergic diseases. AIT often includes depot-forming and immunostimulatory adjuvants, to prolong allergen presentation and to improve therapeutic efficacy. The use of aluminium salts in AIT, which are commonly used as depot-forming adjuvants, is controversially discussed, due to health concerns and Th2-promoting activity. Therefore, there is the need for novel delivery systems in AIT with similar therapeutic efficacy compared to classical AIT strategies. In this study, a triblock copolymer (hydrogel) was assessed as a delivery system for AIT in a murine model of allergic asthma. We show that the hydrogel combines the advantages of both depot function and biodegradability at the same time. We further demonstrate the suitability of hydrogel to release different bioactive compounds in vitro and in vivo. AIT delivered with hydrogel reduces key parameters of allergic inflammation, such as inflammatory cell infiltration, mucus hypersecretion, and allergen-specific IgE, in a comparable manner to standard AIT treatment. Additionally, hydrogel-based AIT is superior in inducing allergen-specific IgG antibodies with potentially protective functions. Taken together, hydrogel represents a promising delivery system for AIT that is able to combine therapeutic allergen administration with the prolonged release of immunomodulators at the same time.

## 1. Introduction

Allergic diseases show an increasing prevalence worldwide, and include several complex multifactorial disorders with varying symptoms and conditions [1,2,3]. In type I hypersensitivity reactions, including atopic diseases such as allergic asthma and rhinitis, common features include an inappropriate immune response to an innoxious environmental antigen, leading to IgE-mediated inflammatory responses and reduced immune tolerance [4,5,6]. The underlying mechanisms are not fully understood yet, leaving allergen-specific immunotherapy (AIT) as the only currently available curative treatment option that is able to modulate the allergic immune response and to restrict disease progression [7,8]. Although showing high efficacy for many patients, some patients do not respond sufficiently to the current treatment protocols [9]. Advanced therapeutic options, and a better understanding of the mechanisms underlying AIT, are necessary to improve therapeutic outcome.

The development of novel therapeutic strategies also includes the identification of immunomodulators, which can positively influence the therapeutic outcome [10,11]. One question arising with the discovery of suitable immunomodulators addresses the route of administration, or more specifically, how the route of administration can influence the efficacy of delivery, as well as the bioavailability [12,13,14,15]. Whereas AIT for patients takes several years, the usage of murine AIT models allows the mimicking of the human situation, with the advantage of gaining deep insights into cellular processes and the generation of rapid results within weeks [16,17,18,19,20].

Most immunomodulators that could be beneficial for the development of new treatment strategies have short half-life periods, resulting in the need of repetitive administration in short intervals to reach adequate efficacy. The utilization of a matrix with depot function for delivery could lead to a prolonged and continuous release of immunomodulators, ideally sparing the patients daily injections. The combined administration of the allergens needed for AIT and the immunomodulator in one treatment could reduce the number of necessary injections even further. Additionally, new delivery strategies could replace aluminium compounds as adjuvants in AIT. Aluminium enhances the immune response by the recruitment and activation of dendritic cells (DCs) [21]. Activated DCs then drive the T helper (Th) cell differentiation towards a Th2 response; therefore, the use of aluminium might counteract the beneficial therapeutic effects of AIT [22,23]. Although controversially discussed, a reason to use aluminium-based adjuvants is their potential depot function [24]. The principle behind AIT is to gradually desensitize the patients to the causative antigen, and redirect the immune response towards a tolerogenic profile [25,26,27,28]. The formed depot of aluminium and antigen results in a local deposition of the allergen with a following gradual release. The prevention of immediate release reduces the risk of anaphylaxis and contributes to the safety of AIT [29]. But the use of aluminium compounds in vaccines is not only beneficial. Side effects regarding toxicity and several health aspects came to attention, and were discussed intensely in several reviews [30,31,32,33]. To avoid possible negative aspects associated with the use of aluminium, hydrogels could be a considerable alternative.

Hydrogels, defined as three-dimensional cross-linked polymer networks, are able to hold a large fraction of aqueous solvents [34,35,36]. They are already shown to be suitable for the release of several substances such as growth hormone or interleukin (IL)-2 [37,38]. This could also be beneficial for the administration of immunomodulators, as well as for the delivery of AIT. Comparable results of AIT by regular subcutaneous injections of antigen and the delivery with hydrogel are a requirement that needs to be fulfilled to realize this strategy.

The hydrogel tested in this study, a triblock copolymer consisting of A-blocks (polyethylenglycol (PEG)) and B-blocks (poly(lactide-co-glycolide) (PLGA)) arranged in a BAB type with a DL-lactide/glycolide molar ratio of 19:1, provides an in situ gel-forming system. Hydrophobic PLGA segments form associative cross-links and the hydrophilic PEG segments allow the copolymer segments to stay in the solution. This aqueous solution is dominated by hydrogen bonding between PEG segments and H_2_O molecules. An increase in temperature results in the weakening of hydrogen bonding, while hydrophobic forces among the PLGA segments gain strength, leading to a transition from solution to gel [39]. It exists in liquid state below body temperature, and quickly converts into a gel after injection. This gel, which degrades slowly over time, then acts as a depot for the delivery of bioactive agents. Thermo-responsiveness and biodegradability make hydrogels an interesting delivery system. In comparison to PLGA microspheres or peptide- and protein-based delivery systems, block copolymers have the advantage of simple compounding, sterilization by filtration, and the use of aqueous solutions as solvents [40,41]. Here we evaluate the use of a PLGA–PEG–PLGA triblock polymer (hydrogel) in AIT. A well-described murine model of AIT, based on ovalbumin (OVA)/aluminium (alum)-sensitization, was used to evaluate the therapeutic efficacy in comparison to subcutaneous injections without hydrogel [42].

## 2. Materials and Methods

### 2.1. Hydrogel Synthesis

PEG 1500 (7.8 g) (Merck, Darmstadt, Germany) was melted at 120 °C for 30 min under stirring. Vacuum was applied, and heating at 120 °C was continued for 2 h. Addition of 16.6 g DL-lactide (abcr, Karlsruhe, Germany) and 0.89 g glycolide (Merck) was performed under a gentle argon stream, with subsequent mixing and melting for 30 min at 120 °C. After addition of 40 µL tin(II)-ethylhexanoate (Merck), polymer synthesis ran at 150 °C for 16 h.

For purification, 25 mL acetonitrile (Roth, Karlsruhe, Germany) was added and mixed at RT until the polymer was completely dissolved. Ultrapure water (200 mL) was added and mixed until a clear solution was obtained. The polymer solution was heated to 80 °C for 45 min to precipitate. Supernatant was removed, and purification steps were repeated two times with 15 mL acetonitrile and 150 mL ultrapure water. The polymer was dissolved in 15 mL acetonitrile, and 25 mL ultrapure water was added after the final purification step. Purified polymer solution (hydrogel) was freeze-dried, and stored at 4 °C.

### 2.2. Determination of Gelling Temperature and Stability of Polymer Solution

Differently concentrated hydrogel solutions were heated at a rate of 1 °C every 5 min. Tube inversion method was used to determine the lowest temperature that induced gel formation. Temperature was further increased after gel formation, and the precipitation temperature was set at the lowest temperature at which the polymer solution started flowing again. A total of 20% of the hydrogel solutions were stored at 4 °C for the duration of the stability study.

### 2.3. Preparation of 20% (w/v) Hydrogel for Injection

Freeze-dried hydrogel was dissolved at 20% (*w/v*) in phosphate-buffered saline (PBS) at 4 °C, and sterile filtrated using a 0.22 µm PVDF membrane filter.

### 2.4. In Vitro Protein Release Assays

A total of 20% of the hydrogel solutions were supplemented with 1 µg polyclonal rabbit anti-lysozyme antibody (Sigma-Aldrich, St. Louis, MO, USA), 20 mM N-acetyl-L-cysteine (NAC) (Sigma-Aldrich, USA), or 0.5 mM sodium salicylate (SA) (Sigma-Aldrich, USA). Loaded hydrogel solutions were incubated at 37 °C until gel formation was completed. The gel was layered with PBS, and incubated at 37 °C while shaking. PBS was collected and replaced with an equivalent amount of fresh PBS after 1, 2, 4, 6, 8, and 24 h for polyclonal rabbit lysozyme antibody, and after 1, 2, 3, 4, 5, 6, 12, and 24 h for NAC and SA.

The amount of polyclonal rabbit anti-lysozyme antibody in the collected supernatants was determined by ELISA. Plates were coated with lysozyme, and released antibodies were detected with alkaline phosphatase-conjugated goat anti-rabbit IgG antibody (Sigma-Aldrich, USA).

NAC concentrations were determined by assays for sulfhydryl groups. Concentrations higher than 15 nM were detected with an assay described by Grassetti and Murray [43], and concentrations below 15 nM were analyzed by an assay described by Kukoc-Modun and Radić [44].

Released SA in the collected supernatants was analyzed by a direct UV assay (TrayCell, Hellma, Müllheim, Germany).

### 2.5. Animals

Female C57BL6/J mice at the age of 5–6 weeks (Charles River, Sulzfeld, Germany) were housed under specific pathogen-free conditions, in individually ventilated cages equipped with wood chips and nesting material. Mice were provided with a standard chow diet and water ad libitum. For the MRI experiments, female, hairless SKH1 mice at the age of 13 weeks (Charles River, Germany), housed under the same conditions, were used. All experiments were approved by the government of the district of upper Bavaria, Germany (ethical approval: 55.2-1-54-2532-50-2017 and 55.2-1-54-2532-104-13), and carried out under the federal guidelines for the use and care of laboratory animals.

### 2.6. MR Imaging

For monitoring in vivo hydrogel degradation, female SKH1 mice (*n* = 2) were injected with 50 µL or 100 µL hydrogel; animals were imaged right after injection and on days 6, 12, and 21 using an MRI scanner (mice were aged to at least 15 months old before being sacrificed).

For monitoring protein release, female SKH1 mice (*n* = 2) were injected with 100 µL of hydrogel containing 1 mg and 5 mg gadolinium-labelled albumin (Galbumin^TM^ BioPal, Worcester, MA, USA), and animals were imaged right after injection and then 8 days later (mice were aged to at least 4 months old before being sacrificed). For calibration, a cylindrical phantom containing hydrogel/gadolinium-labelled albumin was positioned close to the mouse.

All MRI measurements were conducted using a 9.4 T Biospec 94/20 USR (Bruker BioSpin GmbH, Ettlingen, Germany) small animal system, equipped with a 675 mT/m gradient system B-GA12S HP (Bruker BioSpin GmbH). For signal acquisition, a volume resonator with an inner diameter of 72 mm (Bruker BioSpin GmbH) was used. The mice were anaesthetized by isoflurane inhalation, initiated with 5%, and then sustained with 1–3% isoflurane in 100% oxygen. Anaesthetized animals were placed in the supine position and covered by a warming cover, which was connected to a heating circuit and maintained at 37 °C. A small animal physiological monitoring system (SA Instruments, Stony Brook, NY, USA) was used to monitor respiration rate by a pressure-sensitive pad, and body temperature by a rectal thermometer. At each time point, two axial data sets, T1-weighted and T2-weighted data, covering a region of approximately 50 mm, were acquired. The T1 images were obtained using the following parameters: 2D FLASH sequence; repetition time (TR) 700 ms; echo time (TE) 2.5 ms; excitation pulse angle 70°; field of view (FOV) 30 × 30 mm²; acquisition matrix 256 × 256; slices 40; slice thickness 0.5 mm; interslice distance 0.7 mm; bandwidth 69,444.4 Hz; averages 1. The T2 data were acquired using the following parameters: 2D RARE sequence; repetition time (TR) 4362 ms; echo time (TE) 33 ms; pulse angle 90°/180°; FOV 30 × 30 mm²; acquisition matrix 256 × 256; slices 40; slice thickness 0.5 mm; interslice distance 0.7 mm; bandwidth 41,666.7 Hz; averages 8.

### 2.7. Murine Model of AIT

Female C57BL6/J mice were divided into five groups (*n* = 10). The non-allergic and non-allergic–hydrogel groups represented non-allergic, non-sensitized control groups. The non-allergic–hydrogel group was injected with unloaded hydrogel to exclude any effects of hydrogel itself. The allergic group represented allergic, untreated individuals, whereas the AIT group included allergic mice that underwent the standard AIT treatment. Mice in the AIT–hydrogel group were allergic, and received AIT treatment delivered in hydrogel.

Allergic mice and mice of the AIT groups were sensitized via intraperitoneal (i.p.) injections on days 1, 7, and 21 with 30 µg of ovalbumin grade V (OVA) (Merck) and 2 mg of aluminum hydroxide (alum) (Thermo Fisher Scientific, Waltham, MA, USA) in 200 µL PBS. Non-allergic mice received injections of 2 mg alum in PBS (i.p.). After sensitization, the AIT group without hydrogel was treated via subcutaneous (s.c.) injections of 500 µg OVA in 200 µL PBS on days 35, 38, 41, and 44; the AIT group with hydrogel received 500 µg OVA in 200 µL hydrogel. Non-allergic mice were treated with 200 µL PBS or 200 µL hydrogel (s.c.), whereas allergic mice were treated with 200 µL PBS only. All mice were challenged with 1% nebulized OVA for 15 min on days 57, 60, and 63, and euthanized on day 64.

### 2.8. In Vivo Release Assay

Two groups of female C57BL6/J mice (*n* = 3) were injected with either 100 µg IL-4 mutein (IL-4M) in PBS (i.p.), or with 100 µg IL-4M in 200 µL of 20% hydrogel loaded (s.c.). Blood was sampled 24 h after injection, and IL-4M concentrations were analyzed in serum samples. IL-4M was generated as previously described [42].

### 2.9. Isolation of BALF Cells and Lung Lymphocytes

Bronchioalveolar lavage (BAL) and isolation of cells from the bronchoalveolar lavage fluid (BALF) was performed as previously described [42]. Cytokine levels in the BALF were determined using BioLegend’s LEGENDplex T Helper Cytokine Panel (BioLegend, San Diego, CA, USA), according to the manufacturers’ instructions. Lung lymphocytes were isolated as previously described [42].

### 2.10. Flow Cytometry

Isolated BALF or lung tissue cells were stained with a fixable viability dye for 10 min at 4 °C to exclude dead cells. Cells were washed twice with FACS buffer (1% FCS in PBS), and labeled with antibodies against surface markers for 20 min at 4 °C. All antibodies used are listed in Tables S1 and S2. Next, cells were washed twice and subsequently fixed with eBioscience^TM^ FoxP3/Transcription Factor kit (Thermo Fisher Scientific) for 30 min. After washing twice with fixation/permeabilization kit buffer, intracellular staining was performed for 1 h at 4 °C. Cells were washed once with fixation/permeabilization kit buffer and FACS buffer, resuspended in FACS buffer, and analyzed using a BD LSRFortessa™ flow cytometer, BD FACSDiva software version 8.0.1 (BD Biosciences, San Jose, CA, USA) and FlowJo V.7.2.2 (Tree star, Ashland, OR, USA).

### 2.11. Measurement of Immunoglobulins

Total IgG1 (tIgG1) levels were analyzed using the LEGENDplex™ Mouse Immunoglobulin Isotyping Panel (BioLegend), according to the manufacturer’s instructions.

For the detection of OVA-specific IgG1 (OVA-sIgG1), plates were coated with 1 µg/mL ovalbumin grade V (Merck). OVA-sIgG1 was detected with a biotinylated anti-mouse IgG1 antibody (BD Biosciences).

Quantification of total IgE (tIgE) was performed with the BD Mouse IgE ELISA set (BD Biosciences), following the manufacturer’s instructions.

OVA-specific IgE (OVA-sIgE) was analyzed using the LEGEND MAX™ Mouse OVA Specific IgE ELISA Kit (BioLegend), according to the manufacturer’s instructions.

### 2.12. Histological Analysis

Lungs were excised after bronchoalveolar lavage (BAL). The left lobe was fixed in 4% buffered formalin and embedded in paraffin. Sections of 4 µm thickness were stained with hematoxylin–eosin (H&E) and periodic acid Schiff (PAS). Mucus hypersecretion and inflammatory cell infiltration were graded in a blinded fashion, on a scale from 0 to 4 (0 = none, 1 = mild, 2 = moderate, 3 = marked, and 4 = severe), reflecting the degree of the pathological alteration of each airway [45].

### 2.13. Statistical Analysis

GraphPad Prism6 (GraphPad Software, La Jolla, CA, USA) was used to perform the statistical analysis. Specifically, Gaussian distribution was tested by D’Agostino–Pearson omnibus normality test. Gaussian and non-Gaussian distributed results were further analyzed by unpaired *t*-test or Mann–Whitney test, respectively. For analysis of inflammatory cell infiltrate and mucus hypersecretion, Gaussian distributed results were analyzed by one-way ANOVA with Tukey’s test. *p*-values of ≤0.05, ≤0.01, ≤0.001, and ≤0.0001 are shown as *, **, ***, and ****, or +, ++, +++, and ++++, respectively.

GraphPad Prism6 (GraphPad Software, La Jolla, CA, USA) was used to perform the statistical analysis. Specifically, Gaussian distribution was tested by D’Agostino–Pearson omnibus normality test. Gaussian and non-Gaussian distributed results were further analyzed by unpaired *t*-test or Mann–Whitney test, respectively. For analysis of inflammatory cell infiltrate and mucus hypersecretion, Gaussian distributed results were analyzed by one-way ANOVA with Tukey’s test. *p*-values of ≤0.05, ≤0.01, ≤0.001, and ≤0.0001 are shown as *, **, ***, and ****, or +, ++, +++, and ++++, respectively.

## 3. Results

### 3.1. Hydrogel Forms a Solid Depot and Is Biodegradable In Vivo

**Figure 1:**
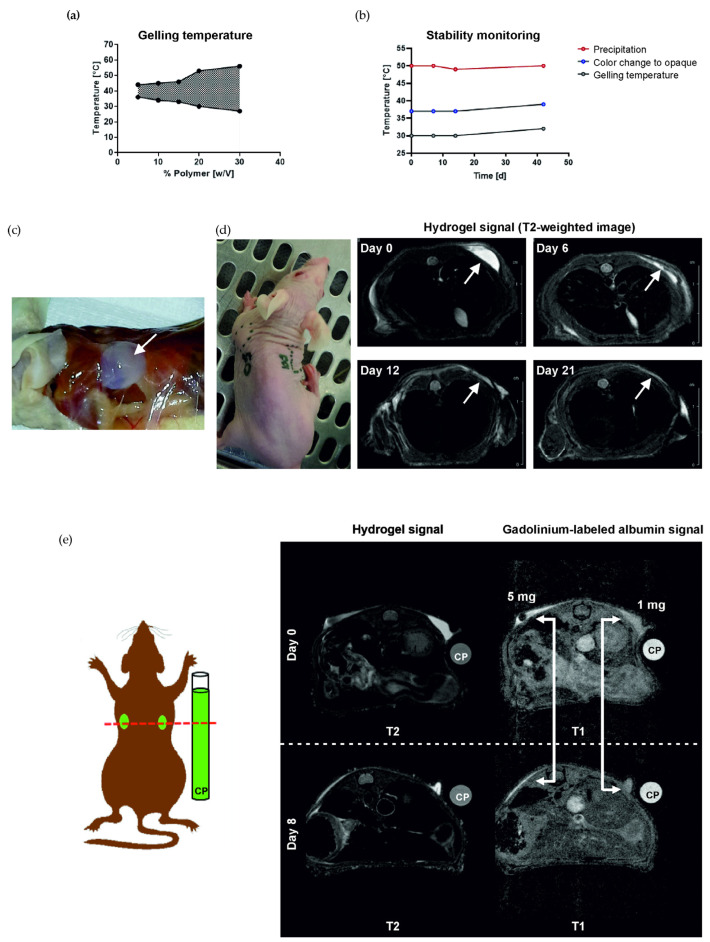
Stability monitoring, depot formation, and biodegradability of hydrogel. (**a**) Gelling behavior of hydrogel solutions with a polymer content of 5, 10, 15, 20, and 30% (*w/v*). (**b**) Stability monitoring of hydrogel over a period of 6 weeks. (**c**) Depot formation 2 h after injection of 100 µL hydrogel (s.c.). (**d**) MRI monitoring of the degradation of a 100 µL hydrogel depot. Images were taken directly after injection as well as on days 6, 12, and 21. (**e**) MRI monitoring of gadolinium-labeled albumin, injected with hydrogel directly after injection and on day 8. White arrows indicate the location of the hydrogel depot. CP, cylinder phantom with hydrogel loaded with gadolinium-labeled albumin; T1, T1-weighted; T2, T2-weighted.

To analyze the gelling behavior and determine the optimal polymer concentration for in vivo administration, concentrations of 5, 10, 15, 20, and 30% polymer in PBS (*w/v*) were studied at different temperatures (Figure 1a). An increase in polymer content results in a larger temperature range, in which the polymer solution is in a solid state. All further experiments were performed with 20% polymer in PBS (*w/v*). For this concentration, the polymer solution is in liquid state at room temperature and becomes solid at 32 °C. Therefore, it could easily be applied at RT, keeping the liquid state below 32 °C, and turning to gel quickly after administration. The polymer has a number average molar mass of 5669 g/mol, a mass average molar mass of 7118 g/mol, a polydispersity index of 1.256, and a lactide to glycolide ratio of approx. 19:1 (data not shown).

The stability of the 20% polymer solution in PBS (*w/v*) was assessed at different time points (Figure 1b). Evaluation of gelling temperature, color change from translucent to opaque after gel formation, and precipitation temperature was performed on days 0, 7, 14, and 42. No significant changes were measured during this time course, only a trend towards an increase in the measured parameters after 42 days. For all further studies, hydrogel was dissolved and used within one week.

Depot formation of 20% hydrogel was also confirmed in vivo. Dissection 2 h after the injection of 100 µL hydrogel (s.c.) shows a clearly delimited depot (Figure 1c). MR imaging of injected hydrogel shows depot formation directly after injection, and degradation over a time course of 21 days (Figure 1d). The injection of hydrogel loaded with 1 mg or 5 mg gadolinium-labeled albumin could confirm the ability of hydrogel to act as a depot for bioactive compounds.

### 3.2. Hydrogel Is Suitable for In Vitro and In Vivo Release of Bioactive Molecules

**Figure 2:**
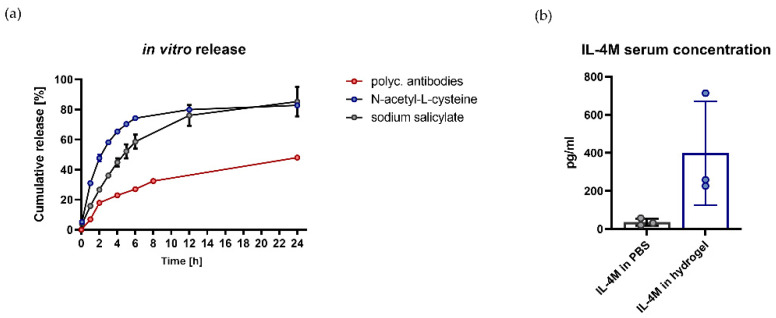
In vitro and in vivo release of bioactive compounds by hydrogel. (**a**) In vitro release of polyclonal anti-lysozyme antibodies, N-acetyl-cysteine, and sodium salicylate by hydrogel, analyzed over a period of 24 h. (**b**) Release of IL-4M administered systemically in PBS (i.p.) or locally (s.c.) in hydrogel. IL4-M concentration was analyzed in serum 24 h after injection (*n* = 3).

The suitability of hydrogel as a depot for various compounds was first tested with in vitro release assays (Figure 2a). Here, a continuous release over a period of 24 h could be demonstrated for a polyclonal rabbit anti-lysozyme antibody NAC and SA, as exemplary bioactive components with different molecular properties.

To address the release characteristics of hydrogel in vivo, mice were injected with 100 µg IL-4M in 200 µL hydrogel (s.c.), or with 100 µg IL-4M in 200 µL in PBS (i.p.). The local administration of IL-4M with hydrogel results in higher concentrations in serum in comparison to the systemic administration of IL-4M in PBS 24 h after the injection (Figure 2b).

### 3.3. Administration of AIT via a Hydrogel Depot Significantly Reduces the Infiltration of BALF Cells and the Production of Th2 Cytokines

**Figure 3:**
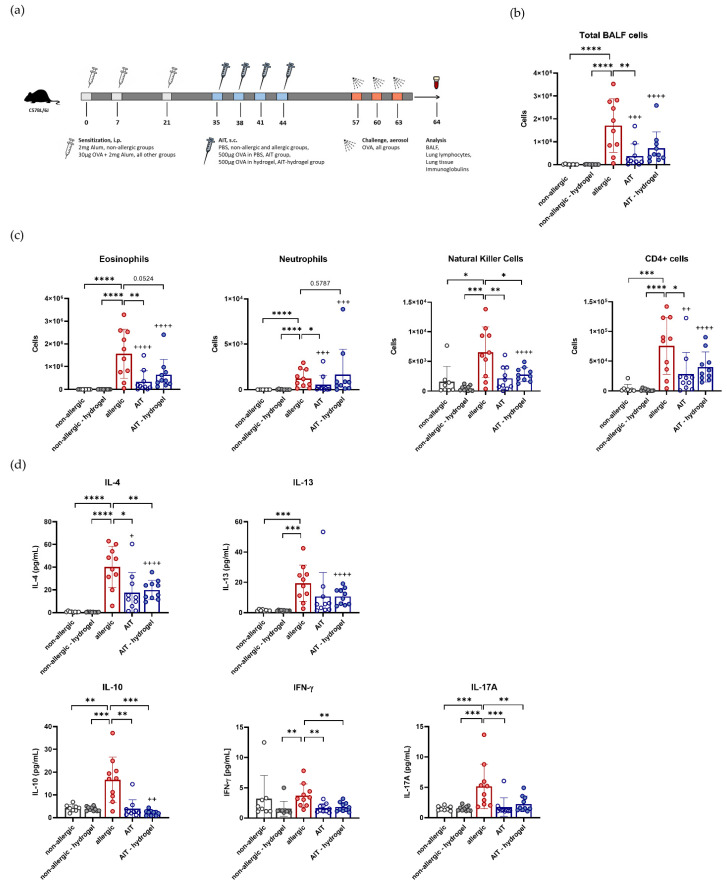
Effects of AIT delivered with hydrogel in comparison to standard AIT treatment on BALF cells and cytokines. (**a**) Schematic overview of AIT in a murine model of allergic asthma. (**b**) Total BALF cell count (*n* = 10). (**c**) Differential cell counts of eosinophils, neutrophils, natural killer cells, and CD4+ cells in the BALF (*n* = 10). (**d**) Levels of IL-4, IL-13, IFN-γ, IL-10, and IL-17A analyzed in the BALF (*n* = 10). Gaussian and non-Gaussian distributed results were analyzed by unpaired t-test or Mann–Whitney test, respectively. *p*-values of ≤0.05, ≤0.01, ≤0.001, and ≤0.0001 are shown as *, **, ***, and ****, respectively. *p*-values of AIT groups compared to the respective non-allergic groups are shown as +, ++, +++, and ++++, respectively.

The efficacy of AIT, administered with or without hydrogel, was compared in a murine model of allergic asthma (Figure 3a). A comparison between the non-allergic groups with and without hydrogel was performed to exclude effects of the hydrogel itself. Injection sites of hydrogel were monitored regularly, and no signs of local reactions (swelling, reddening, or inflammation of the skin) could be detected.

Allergic mice show a significant increase in total BALF cells in comparison to both non-allergic groups. AIT treatment significantly reduces the number of total BALF cells in comparison to the allergic group. For mice treated with AIT in hydrogel, a reduction in total BALF cells is also detected, although not significantly. Both treatment groups do not differ significantly from each other (Figure 3b). Moreover, both therapeutic strategies reduce eosinophils, neutrophils, CD4+ T cells, and natural killer cells in the BALF in a comparable manner (Figure 3c).

Analysis of cytokines in the BALF reveals a comparable, significant reduction in the Th2 cytokine IL-4 for both therapeutic strategies in comparison to the allergic groups, and a trend towards reduced levels of IL-13 (Figure 3d). Additionally, levels of IL-10, IFN-γ, and IL-17A are reduced to a similar extent in both treatment groups, compared to the allergic group.

Comparing the non-allergic groups, with and without hydrogel, there are no differences in cell count or cytokine levels in the BALF (Figure 3b–d). Overall, both therapeutic strategies lead to comparable effects on the analyzed parameters in the BALF.

### 3.4. AIT Delivered with Hydrogel Significantly Reduces Mucus Hypersecretion and Cell Infiltration in Lung Tissue

**Figure 4:**
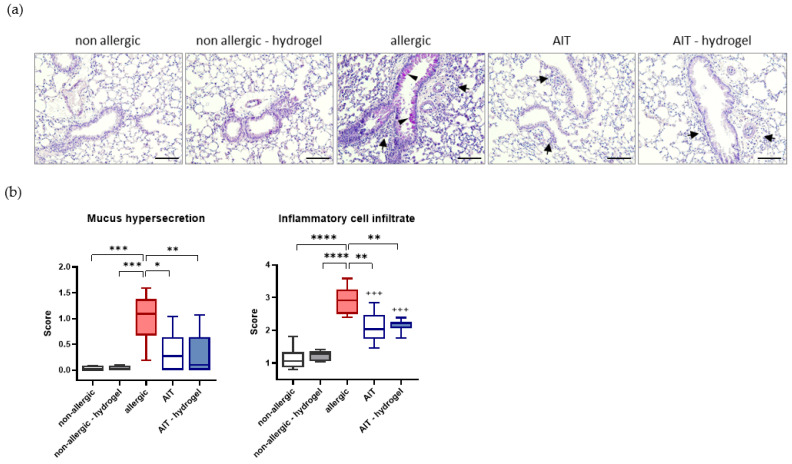
Effects of AIT delivered in hydrogel compared to standard AIT treatment on mucus hypersecretion and infiltration of inflammatory cells in lung tissue. (**a**) Representative lung histology (PAS staining) of non-allergic, non-allergic–hydrogel, allergic, AIT, and AIT–hydrogel groups. Arrows, inflammatory infiltrate; arrowheads, mucus hypersecretion; scale bar: 100 μm. (**b**) Histological scoring of inflammatory cell infiltration and mucus hypersecretion (*n* = 6); boxplots indicate minimum, 25th percentile, median, 75th percentile, and maximum. Results were analyzed by one-way ANOVA with Tukey’s test. *p*-values of ≤0.05, ≤0.01, ≤0.001, and ≤0.0001 are shown as *, **, ***, and ****, respectively. *p*-values of AIT groups compared to the respective non-allergic groups are shown as +++.

Histological analysis of lung tissue shows a significant increase in mucus hypersecretion, determined by PAS staining, and inflammatory cell infiltrate in allergic mice compared to both non-allergic groups (Figure 4a). For both treatment strategies, mucus hypersecretion and inflammatory cell infiltration in the lung tissue are reduced significantly in comparison to allergic mice (Figure 4b).

### 3.5. Effects on Th2 and Treg Cells in Lung Lymphocytes Are Independent of AIT Delivery Strategy

**Figure 5:**
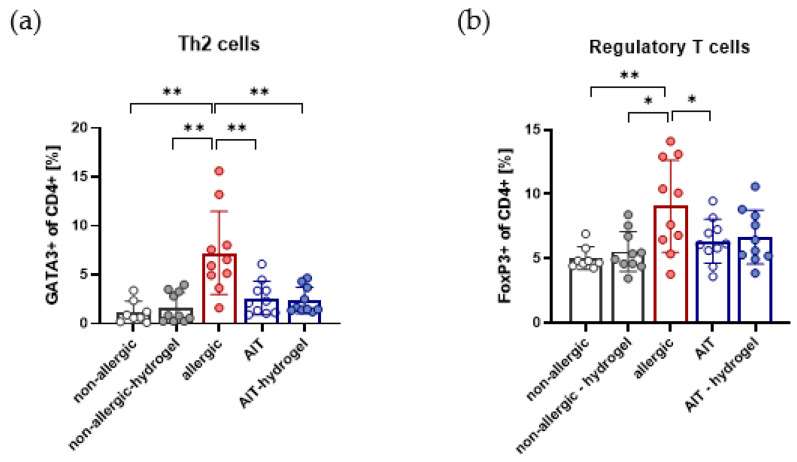
Effects of both AIT treatment strategies on Th2 and regulatory T cells in lung lymphocytes. (**a**) Analysis of pulmonary Th2 cells (*n* = 10). (**b**) Analysis of pulmonary regulatory T cells (*n* = 10). Gaussian and non-Gaussian distributed results were analyzed by unpaired t-test or Mann–Whitney test, respectively. *p*-values of ≤0.05 and ≤0.01 are shown as * and ** respectively.

FACS analysis of lung lymphocytes, isolated from whole lung tissue, shows a significant reduction in GATA3^+^ Th2 cells for both AIT delivery strategies in comparison to allergic mice. No difference in treatment efficiency is detected, regarding the capacity to reduce Th2 cells in lung tissue (Figure 5a). The analyzed FoxP3^+^ population within the CD4^+^ T helper cells, representing regulatory T cells (Tregs), are significantly reduced after AIT compared to the allergic group. Applying AIT in combination with hydrogel leads to a similar, although not significant, reduction in Tregs (Figure 5b). For both non-allergic groups, no differences are detectable. Both treatment strategies exert comparable effects on analyzed CD4^+^ T helper cell populations in lung tissue.

### 3.6. Total IgG1, OVA-Specific IgG1, and OVA-Specific IgE Are Increased in AIT–Hydrogel Treated Mice

**Figure 6:**
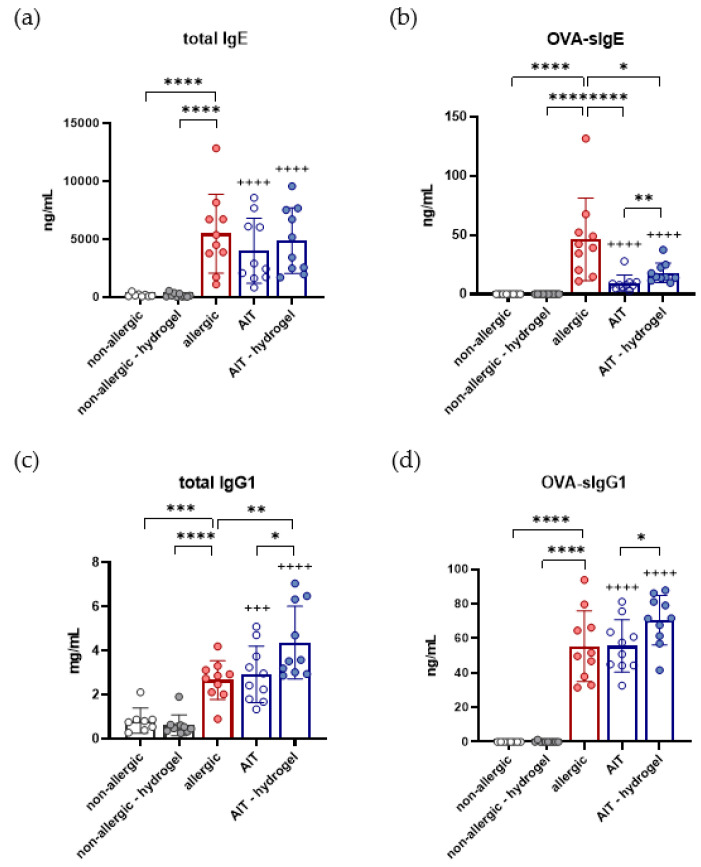
Effects of both AIT treatment strategies on the humoral immune response in a murine model of allergic asthma. Measurement of total IgE (**a**), OVA-sIgE (**b**), total IgG1 (**c**), and OVA-sIgG1 (**d**) in serum samples at the endpoint of the experiment (*n* = 10). Gaussian and non-Gaussian distributed results were analyzed by unpaired t-test or Mann–Whitney test, respectively. *p*-values of ≤0.05, ≤0.01, ≤0.001, and ≤0.0001 are shown as *, **, ***, and ****, respectively. *p*-values of AIT groups compared to the respective non-allergic groups are shown as +++ and ++++, respectively.

Analysis of immunoglobulins at the end of the experiment shows a significant increase in tIgE in the allergic group, as well as both AIT groups, in comparison to non-allergic mice (Figure 6a). Comparable levels of tIgE are detected for allergic and AIT groups. Measurements of OVA-sIgE reveal a significant increase in the allergic group in comparison to both non-allergic groups (Figure 6b). AIT groups show a significant reduction in OVA-sIgE in comparison to the allergic group. Focusing on the two AIT groups, OVA-sIgE levels are significantly reduced in the standard AIT group compared to AIT delivered in hydrogel.

A significant increase in tIgG1 is also observed for the allergic group, as well as for both AIT groups, in comparison to non-allergic mice (Figure 6c). tIgG1 levels do not differ significantly between allergic and standard AIT-treated mice, but a significant increase is detectable for the AIT–hydrogel treatment strategy. Analysis of OVA-sIgG1 shows a similar pattern, with a significant increase for allergic and AIT-treated mice compared to the non-allergic groups (Figure 6d). While there is no significant difference detectable between the OVA-sIgG1 levels of the allergic mice and mice that receive standard AIT, there is a significant increase for the AIT–hydrogel group compared to the standard AIT group.

Overall, for all measured immunoglobulins, no significant differences are detectable for the non-allergic groups. Administration of AIT with hydrogel leads to a significant increase in OVA-sIgE, tIgG1, and OVA-sIgG1 compared to standard AIT treatment.

## 4. Discussion

To investigate the suitability of hydrogel as a matrix in AIT, the efficiency of AIT delivered with and without hydrogel was compared. The applied murine model of OVA-induced allergic asthma leads to the expected type I-mediated allergic responses in the group of allergic mice, characterized by elevated levels of tIgE and sIgE, a pro-allergic type II immune response in lung tissue, and an increase in eosinophilic airway inflammation [46,47].

Both therapeutic strategies show comparable efficacy in the reduction in BALF cells and Th2 cells from isolated lung lymphocytes, compared to the allergic group. Whereas standard AIT treatment seems to have a slightly stronger effect on the reduction in infiltrating cells than AIT–hydrogel treatment, no differences are observed in pulmonary Th2 and Treg cells. A similar reduction in the Th2 cytokines IL-4 and IL-13, measured in the BALF, substantiates the hypothesis that delivery of AIT with hydrogel could be an efficient therapeutic strategy. Histological analysis confirms the effects observed in BALF and lung lymphocytes.

Whereas both therapeutic strategies show good comparability on a cellular level, by the reduction in infiltrating cells as well as lung Th2 cells, some differences are detectable in the analysis of immunoglobulins. None of the applied strategies reduce tIgE significantly in comparison to the allergic group. In human AIT, it can take years of therapy to achieve a reduction in tIgE levels. Therefore, tIgE alone is never used to evaluate the therapeutic success of AIT. The concentration of serum IgE regulates the expression of the high-affinity receptor for IgE (FcεRI) on effector cells [48,49], but the total concentration of IgE does not solely determine the strength of the effector cell release. High-affinity IgE molecules can have a large impact on effector cell activation, and may explain difficulties in correlating tIgE, sIgE, and clinical symptoms [50,51]. In contrast to tIgE, there is a significant reduction in OVA-sIgE for both treatment strategies, compared to the allergic group. Nevertheless, the detected OVA-sIgE is significantly higher in serum samples of the AIT–hydrogel group in comparison to standard treatment. This effect is most likely caused by the prolonged presentation of antigen during AIT, due to the hydrogels’ depot function. Interestingly, this effect is not only observed for OVA-sIgE, but also for tIgG1 and OVA-sIgG1, which might be associated with a protective function [52].

In AIT, the induction of local and systemic IgG- and IgA antibodies, following long-term therapy, is related to the restoration of immune tolerance [51,53,54]. In particular, AIT delivered subcutaneously induces a strong IgG response [55]. IgG blocking antibodies are thought to inhibit IgE-mediated mast cell activation by the interception of allergens before they can bind and cross-link FcεRI-bound IgE, or through the inhibitory receptor FcγRIIb [52,56]. The levels of tIgG in serum samples, analyzed at the end of the experiment, are significantly elevated for allergic mice, as well as for both AIT groups, in comparison to non-allergic mice. While the allergic and standard AIT groups show similar tIgG1 levels, mice in the AIT–hydrogel group show a significantly higher level of tIgG1. The same pattern is observed for the quantification of OVA-sIgG1. This is most likely due to the prolonged presence of the allergen during the therapeutic phase. The human antibody IgG4 is often used as a marker to evaluate tolerance-inducing effects and the success of AIT [57,58,59]. Mice lack IgG4, but murine IgG1 functionally resembles human IgG4, and is thought to be the murine equivalent [60]. Reaching higher levels of sIgG1 could, therefore, contribute to the protective effect of AIT, and display a beneficial feature of hydrogel usage in AIT delivery.

An important criterion for the application of hydrogel in AIT is not only a successful therapeutic outcome, but also the suitability of hydrogel as a delivery matrix. An essential requirement is that the injection of hydrogel alone has no inflammatory effects. A comparison of the non-allergic groups with and without hydrogel depot shows no significant differences, or even tendencies, in any of the analyzed parameters and, therefore, confirms the suitability of hydrogel as a potential delivery matrix for AIT or immunomodulators. The suitability of hydrogel as a delivery matrix could be confirmed by the continuous and gradual release of bioactive compounds with in vitro release assays. The resulting increased IL-4M serum levels following in vivo administration of IL-4M with hydrogel compared to systemic administration strengthens the potential of hydrogel as a delivery matrix.

AIT protocols for the treatment of humans often include aluminium in subcutaneous therapeutic injections. An argument supporting the use of aluminium adjuvants in this context is their depot function. The slower release of allergens is thought to improve tolerability towards the allergens, especially in subcutaneous immunotherapy (SCIT) [61]. Prolonged immunization also leads to the induction of allergen-specific IgG, which eventually dominates the IgE response [29,62]. While those aspects may be in favor of aluminium adjuvant usage in AIT, aluminium is also considered to act as a stimulator of the Th2 response [63], namely, the response AIT is actually trying to dampen and modify. Paradoxically, in AIT, a Th2-type disease is treated with a Th2-biasing adjuvant [29]. This leads to the question if therapeutic outcome could be improved by avoiding a boost to this response [64]. A depot-matrix-like hydrogel could offer a considerable alternative, and help to keep the benefits of prolonged immunization by slow allergen release, and avoid additional stimulation of the Th2 response at the same time.

Aluminium salts are widely used and accepted by regulatory authorities in European countries [65]. Nevertheless, more and more health concerns arose during the past few years, opening debates, and leading to demands for considerable alternatives [31,33]. In contrast to prophylactic vaccinations, AIT requires numerous injections with aluminium over several years and, therefore, adds to the aluminium burden of the body [66]. Upon injection, aluminium phagocytizes, which leads to the activation and recruitment of inflammatory cells [67]. There are also studies available that deal with the production of ROS and, therefore, DNA damage caused by an increased aluminium burden [68]. An aluminium-free approach for AIT could avoid possible health concerns arising with an increased aluminium burden. In AIT, microcrystalline tyrosine (MCT) has become a considerable and often used alternative. Similar to aluminium, MCT acts as a depot, and has the ability to enhance the immune response, but is fully metabolized within the body and does not accumulate [69,70]. Nevertheless, establishing an adjuvant-free delivery system for AIT could be beneficial, and help to avoid adverse effects of the adjuvants.

## 5. Conclusions

In this study, we demonstrate that successful AIT can be performed in a murine model of allergic asthma, with the application of hydrogel as delivery matrix. It is evident that hydrogel needs to be carefully tested, regarding the treatment efficacy in comparison to conventional AIT in humans. Further testing is also required to investigate potential limitations of the application regarding size and loading capacity of hydrogel depots in AIT, as well as the translation from animal studies to the clinics. Those studies will give initial indications how the use of a depot matrix could affect current treatment protocols. Nevertheless, this is the first study to show that hydrogel could provide an alternative to achieve the prolonged presence of allergens without the health concerns that arise with the use of aluminium. With respect to the rising health concerns, the successful demonstration of the suitability of hydrogel for AIT opens new possibilities for AIT protocols. In addition, the use of hydrogels introduces the possibility of combined treatment strategies. Hydrogels can be loaded with a variety of substances, and can be injected at the site of interest directly. Loading hydrogel not only with the amount of allergen required for AIT, but also with immunomodulators and/or chemoattractants, could be beneficial for the therapeutic outcome. For example, such an approach could help to increase the uptake of the immunomodulators by recruiting target cells directly to the site of immunomodulator release, with the help of an adequate chemoattractant. Taken together, hydrogels could be a considerable alternative for further studies, investigating different options of the safe and efficient delivery of AIT.

## Data Availability

All data generated or analyzed during this study are included in this article and its Supplementary Material files.

## Supplementary Materials

The following supporting information can be downloaded at: https://www.mdpi.com/article/10.3390/pharmaceutics14081527/s1, Figure S1: Structure of hydrogel; Table S1: Antibodies for FACS analysis of BALF cells; Table S2: Antibodies for FACS analysis of lung lymphocytes.
