# Supplementary material for: Thermosensitive PLGA–PEG–PLGA Hydrogel as Depot Matrix for Allergen-Specific Immunotherapy
Source: Pharmaceutics. 2022 Jul 22;14(8):1527. doi: 10.3390/pharmaceutics14081527 (PMC9329805; doi:10.3390/pharmaceutics14081527)
Supplement: Supplementary file 1 [file pharmaceutics-14-01527-s001.zip › pharmaceutics-1810441-supplementary.pdf]

Supplemental Figures

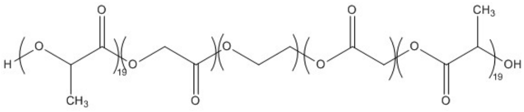

Figure S1. Structure of hydrogel: A triblock copolymer, consisting of A-blocks (polyethylenglycol (PEG)) and B-blocks (poly(lactide-co-glycolide) (PLGA)) arranged in a B-A-B type.

Supplemental Materials

Table S1. Antibodies for FACS analysis of BALF cells.

Marker	Fluorochrome	Clone	Manufacturer
CD4	AF700	RM4-5	BioLegend
CD8	FITC	53-6.7	BD Biosciences
CD11b	BV711	M1/70	BioLegend
CD11c	PE-Cy7	N418	Biolegend
CD45	PerCP-Cy5.5	30-F11	BioLegend
CD206	PE-Dazzle594	C068C2	BioLegend
F4/80	APC-eF780	BM8	eBiosciences
NK1.1	APC	PK136	eBiosciences
Ly-6G	PacificBlue	1A8	Biolegend
Siglec-F	PE	E50-2440	BD Biosciences
Live/dead aqua fixable cell stain	405nm	—	ThermoFisher

Table S2. Antibodies for FACS analysis of lung lymphocytes.

Marker	Fluorochrome	Clone	Manufacturer
CD3ε	FITC	145-2C11	BD Biosciences
CD4	AF700	RM4-5	BioLegend
CD45	APC-eF780	30-F11	eBiosciences
FoxP3	PerCP-Cy5.5	FJK-16s	eBiosciences
GATA3	eF660	TWAJ	eBiosciences
Live/dead aqua fixable cell stain	405nm	—	ThermoFisher
